# Hydrogenated fat diet intake during pregnancy and lactation modifies the PAI-1 gene expression in white adipose tissue of offspring in adult life

**DOI:** 10.1186/1476-511X-7-13

**Published:** 2008-04-04

**Authors:** Luciana P Pisani, Claudia M Oller do Nascimento, Allain A Bueno, Carolina Biz, Kelse T Albuquerque, Eliane B Ribeiro, Lila M Oyama

**Affiliations:** 1 Department of Physiology, Division of Nutrition Physiology, São Paulo Federal University – UNIFESP, Rua Botucatu, Vila Clementino, São Paulo 04023-062, Brazil; 2 Department of Bioscience, São Paulo Federal University – UNIFESP, v. Ana Costa, Santos, São Paulo 11060-001, Brazil

## Abstract

We examine whether feeding pregnant and lactating rats hydrogenated fats rich in *trans *fatty acids modifies the plasma lipid profiles and the expression of adipokines involved with insulin resistance and cardiovascular disease in their 90-day-old offspring. Pregnant and lactating Wistar rats were fed with either a control diet (C group) or one enriched with hydrogenated vegetable fat (T group). Upon weaning, the male pups were sorted into four groups: CC, mothers were receiving C and pups were kept on C; CT, mothers were receiving C and pups were fed with T; TT, mothers were receiving T and pups were kept on T; TC, mothers were receiving T and pups were fed with C. Pups' food intake and body weight were quantified weekly and the pups were killed at day 90 of life by decapitation. Blood and carcass as well as retroperitoneal, epididymal, and subcutaneous white adipose tissues were collected. Food intake and body weight were lower in TC and TT, and metabolic efficiency was reduced in TT. Offspring of TT and TC rats had increased white adipose tissue PAI-1 gene expression. Insulin receptor was higher in TT than other groups. Ingestion of hydrogenated vegetable fat by the mother during gestation and lactation could promote deleterious consequences, even after the withdrawal of the causal factor.

## Introduction

Nutritional conditions during gestation have a major role in the metabolic and hormonal interactions between the maternal body, placenta, and fetus. The fetus growth is influenced by the maternal nutritional condition. Fetal insulin and insulin growth factor (IGF) have major roles in the regulation of growth, and respond rapidly to changes in fetal nutrition [1].

During gestation, changes in the maternal metabolism occur in order to supply nutrition to the fetus. Lipids play a fundamental role in fetal development. Although lipids transfer through placenta is very limited, changes in dietary fatty acids can lead to implications in fetal and postnatal development [2].

Metabolic programming occurs during the period of intra-uterus development. Inadequate nutritional and environmental factors may modify the fetal metabolic programming which could be reflected as deleterious consequences in adulthood, such as predisposition to develop cardiovascular and metabolic diseases [3-5].

Albuquerque et al [6] suggest that the exposition to *trans *fatty acids (TFA) during gestation and lactation can have harmful consequences to the offspring in adulthood. The levels of TFA in maternal milk are directly correlated to the maternal diet during gestation and lactation [7].

The chronic ingestion of hydrogenated vegetable fat rich in TFA and saturated fatty acids modifies the serum lipid profile, increases the risk of cardiovascular diseases, and reduces insulin sensitivity, leading to type 2 diabetes [8-10]. Also, Ibrahim et al [10] demonstrated that treatment with TFA has a much greater effect in decreasing adipocyte insulin sensitivity than treatment with saturated fatty acids. This result was in part explained by a reduction of plasma membrane fluidity in the rats treated with TFA.

Increases in PAI-1 serum concentrations are related to insulin resistance [11,12] and the incidence of cardiovascular diseases in obesity [13-16].

Several studies in the literature have reported decreased adiponectin serum levels in patients with insulin resistance and type 2 diabetes mellitus, obesity and heart disease [17,18]. It has been demonstrated that adiponectin reduces hepatic production of glucose and the concentration of triglycerides in the muscles, thus ameliorating insulin sensitivity [19].

On the other hand, only a few studies have described the effects of the ingestion of TFA during gestation and lactation on the metabolism of offspring in adulthood. In this study we investigated the effects on 90-day-old offspring of maternal ingestion of a diet enriched with partially hydrogenated vegetable oil, rich in TFA, from the beginning of gestation through lactation, with continued exposure after weaning until the 90th day of life. The effects of this diet were also studied when the offspring were exposed to this diet during gestation and lactation only, or just after weaning. Some parameters of the metabolism were analyzed, as well as the gene expression of adipokynes involved with insulin resistance and cardiovascular diseases.

## Methods

### Animals and treatments

The Experimental Research Committee of the São Paulo Federal University approved all procedures for the care of the animals used in this study. Rats were kept under controlled conditions of light (12:12 h light-dark cycle with lights on at 07:00) and temperature (24 ± 1°C). Three-month-old female Wistar rats were left overnight to mate, and copulation was verified the following morning by the presence of sperm in the vaginal smears. On the first day of gestation, rats were isolated in individual cages and randomly divided into two groups, receiving either a control diet (C) or a diet enriched with hydrogenated vegetable fat (T). The diets were maintained throughout gestation and lactation. On the day of delivery, considered as day zero of lactation, each mother was given eight male pups. On the 21st day of life the animals were weaned and the pups were divided in four groups: CC, mothers fed C diet and pups fed C diet; CT, mothers fed C diet and pups fed T diet; TT, mothers fed T diet and pups fed T diet; TC, mothers fed T diet and pups fed C diet.

Both diets were prepared according to the recommendations of the American Institute of Nutrition (AIN-93 G and M) [20], being similar in calories and lipid content. The source of lipids for the C diet was soybean oil, and the principal source for the T diet was partially hydrogenated vegetable fat, rich in TFA. The centesimal composition of the diets is presented in Table 1. The fatty acid profile of each diet, presented in Table 2, was analyzed in the Laboratory of Food Composition, Federal University of Rio de Janeiro (RJ, Brazil), using gas chromatography.

**Table 1:** Composition of the control diet and diet enriched with *trans *fatty acids according to AIN-93

	**Diet (g/100 g)**	
**Ingredient**	**Control**	***Trans***

Casein*	20 (14)	20
L-cystine^†^	0.3 (0.18)	0.3
Cornstarch^†^	62 (71.1)	62
Soybean oil^‡^	8 (5)	1
Hydrogenated vegetable fat^$^	-	7
Butylhydroquinone^†^	0.0014 (0.0008)	0.0014 (0.0008)
Mineral mixture^§^	3.5	3.5
Vitamin mixture^#^	1.0	1.0
Cellulose^†^	5.0	5.0
Choline bitartrate^†^	0.25	0.25
Energy (kcal/g)	4.0 (3.8)	4.0 (3.8)

The first number refers to the growth diet (AIN-93G) and the number in parentheses refers to maintenance diet (AIN-93M) when its composition differed from that of the growth diet. *Casein was obtained from CoperLab, São Paulo, Brazil. ^†^L-cystine, cornstarch, butylhydroquinone, cellulose and choline bitartrate were obtained from Viafarma, São Paulo, Brazil. ^‡^Oil was supplied from soybean (Lisa/Ind. Brazil). ^$^Hydrogenated vegetabke fat was supplied from Unilever, São Paulo, Brazil. ^£^Mineral mix 9mg/kg diet): calcium, 5000; phosphorus, 1561; potassium, 3600; sodium, 1019; chloride, 1571; sulfur, 300; magnesium, 507; iron, 35; copper, 6.0; manganese, 10.0; zinc, 30.0; chromium, 1.0; iodine 0.2; selenium, 0.15; fluoride, 1.00; boron, 0.50; molybdenum, 0.15; silicon, 5.0; nickel, 0.5; lithium, 0.1; vanadium, 0.1 (AIN-93G mineral mix DYETS 210025, Dyets Inc., Bethlehem, PA, USA). ^#^Vitamin mix (mg/kg diet): thiamin HCL, 6.0, riboflavin, 6.0; pyridoxine HCL 7.0; niacin, 30.0; calcium pantothenate, 16.0; folic acid, 2.0; biotin, 0.2; vitamin B12, 25.0; vitamin A palmitate 4000 IU; vitamin E acetate, 75; vitamin D3, 1000 IU; vitamin KI, 0.75. (AIN-93G, vitamin mix, DYETS 310025, Dyets Inc., Bethlehem, PA, USA)

**Table 2:** Fatty acid composition of the diets

	Percentage of total fatty acids	
	
Fatty acid	Control	*Trans*
C14:0	0.60	0.85
C16:0	12.88	15.70
C18:0	3.88	13.77
C14:1	0.49	0.60
C18:1 n-9 *trans*	ND	11.62
C18:1 n-9 *cis*	22.45	24.15
C18:2 n-6 *trans*	ND	0.43
C18:2 n-6 (linoleic)	52.90	24.59
C18:3 n-3(α-linolenic)	5.41	1.51
Total SFA	17.36	30.32
Total MUFA-*cis*	22.94	24.75
Total PUFA-*cis*	58.31	26.10
Total TFA	ND	12.05
PUFA:SFA	3.35	0.86
n-6:n-3	9.78	16.28

SFA, saturated fatty acids; MUFA, monounsaturated fatty acids; PUFA, polyunsaturated fatty acids; ND, not detected, TFA, *trans *fatty acids.

### Fatty acid composition of the diet

For lipid extraction, saponification, and fatty acid methylation, 500 mg of the prepared diets were treated with 2.0 ml of methanol:benzene (4:1, v/v) followed by 200 μl of acetyl chloride under light agitation. Fatty acid methyl esters were separated (SP2560 column, Supelco, Bellefonte, PA, USA) and quantified by gas-liquid chromatography with an ionizable flame detector (Perkin, Wellesley, MA, USA) and hydrogen as the carrier gas. Injection and detection temperatures were 260°C and 280°C, respectively. The run temperature started at 135°C and increased up to 195°C, with a run time of 45 minutes, as described by Albuquerque et al [6].

### Experimental procedures

The offspring were weighed weekly from days 21 to 90 of life, when they were killed by decapitation. Trunk blood was collected and immediately centrifuged. Serum was separated and stored at -70°C for later determination of triglycerides, cholesterol, HDL-cholesterol, glucose, insulin, adiponectin, and leptin. The retroperitoneal (RET), epididymal (EPI) and subcutaneous (SUB) white adipose tissues were partially dissected, immediately frozen in liquid nitrogen, stored at -70°C, and used for quantification of adiponectin and PAI-1 mRNA. Leftover fat depots were immediately immerged in adequate buffer for total protein extraction, to allow quantification of insulin receptor.

### Carcass lipid and protein content

A further group of CC, CT, TC, and TT rats was used for the determination of the carcass lipid and protein content. Carcasses were eviscerated, weighed, and stored at -20°C. Lipid content was measured as described by Stansbie et al [21] and standardized using the method described by Oller do Nascimento and Williamson [22]. Briefly, the eviscerated carcass was autoclaved at 120°C for 90 minutes and homogenized with double the mass of water. Triplicate aliquots of this homogenate were weighed and digested in 3 ml of 30% KOH and 3 ml of ethanol for at least 2 hours at 70°C in capped tubes. After cooling, 2 ml of 12N H_2_SO_4 _were added and the sample was washed three times with petroleum ether for lipid extraction. Results are expressed as grams of lipid per 100 g of carcass. For protein measurements, aliquots of the same homogenate (approximately 1 g) were heated to 37°C for 1 hour in 0.6N KOH with constant shaking. After clarification by centrifugation, protein content was measured according to the method described by Lowry et al [23].

### RNA extraction and Northern blot analysis

Total RNA was extracted with Tri-Reagent (Sigma), and its concentration was determined from absorbance at 260 nm.

Adiponectin mRNA from RET was quantified by Northern blot analysis, using an antisense oligonucleotide (5'-GTTGCAGTGGAATTTGCCAGTGCCGTCA-3) based on the rat adiponectin cDNA sequence (PubMed), synthesized as a hybridization probe and end-labeled (5'-end) with a digoxigenin ligand (MWG, USA), as described by Trayhurn et al [24].

Twenty-microgram aliquots of RNA were run in 1% agarose-formaldehyde gel at 80–100 V, blotted overnight onto a positively charged nylon membrane (Roche), cross-linked under UV light, pre-hybridized with hybridization buffer (5 × SSC, 50 mM sodium phosphate, 2% blocking reagent, 0.1% N-lauroylsarcosine, 7% SDS, all from Sigma), and hybridized overnight at 42°C with the same buffer with an added rat adiponectin probe at a 25 ng/ml final concentration. Following post-hybridization washes, membranes were incubated with an antibody against digoxigenin (Fab-fragment; Roche) for 30 minutes and then with CDP-Star chemiluminescence substrate (Roche) for 10 minutes at room temperature. Signals were collected by exposure to film for 5–30 minutes at room temperature. After probing for adiponectin mRNA, blots were stripped and re-probed for 18S rRNA. The sequence of the antisense oligonucleotide 18S rRNA was described previously [25]. Blots were quantified by densitometry using Image J software. Results are presented as the mRNA/18S rRNA ratio and expressed as arbitrary units.

### Real-time polymerase chain reaction

PAI-1 mRNA from RET and EPI was quantified by real-time polymerase chain reaction (PCR). RNA samples were previously treated with DNAse (DNA-free, Ambion, UK). One microgram of each sample was reverse transcribed using an M-MLV Reverse Transcriptase kit (Promega), and cDNA was synthesized in a final volume of 50 μl. The relative level of PAI-1 mRNA was quantified in real time, using a SYBR Green primer in an ABI Prism 7700 Sequence Detector (both from Applied Biosystems). The relative level of the housekeeping gene hypoxanthine phosphoribosyltransferase (HPRT) was measured. The primers used were: PAI-1, 5'ACAGCCTTTGTCATCTCAGCC3' (sense) and 5'CCGAACCACAAAGAGAAAGGA3' (antisense); and HPRT, 5'CTCATGGACTGATTATGGACAGGA3' (sense) and 5'GCAGGTCAGCAAAGAACTTATAGC3' (antisense).

Results were obtained using Sequence Detector software (Applied Biosystems) and are expressed as a relative increase, using the method of 2^-ΔΔCt ^described by Livak and Schmittgen [26].

### Quantification of insulin receptor by Western blotting

Samples of EPI and SUB were rapidly dissected and homogenized in 1 ml of chilled extraction buffer (100 mM Trizma Base pH 7.5; 10 mM EDTA; 100 mM NaF; 10 mM Na_4_P_2_O_7_; 10 mM Na_3_VO_4_; 2 mM PMSF; 0.1 mg/ml aprotinin). After homogenization, 100 μl of 10% Triton X-100 was added to the samples, which were left in ice for 30 minutes and then centrifuged (12,000 rpm, 20 minutes, 4°C). From each sample, 80 μg of protein in a 20 μl volume were loaded into a polyacrylamide gel containing 0.1% SDS, separated by electrophoresis, and then electroblotted onto a nitrocellulose membrane. Membranes were incubated with rabbit anti-rat primary antibody against insulin receptor (SC-711), followed by washings and incubation with goat anti-rabbit IgG-horseradish peroxidase (Sigma, USA). The labeled protein was detected by the chemiluminescent substrate ECL (Amersham) and exposed to Hyper-film ECL (Amersham). The protein bands were identified according to their migration in comparison to the rainbow recombinant protein molecular weight markers and quantified by densitometry using Image J software. Membranes were treated with specific buffers to remove previously linked antibodies, incubated with anti-β actin, and measured again. Results are expressed as arbitrary units, relative to β-actin values.

### Biochemical and hormonal serum analysis

Glucose, triglycerides, total cholesterol, and HDL-cholesterol serum concentrations were measured by an enzymatic colorimetric method using commercial kits (Labtest, Brazil). Insulin, adiponectin, and leptin concentrations were quantified using specific enzyme-linked immunosorbent assay (ELISA) kits (Linco Research, USA).

### Statistical analysis

All results are presented as means ± standard error of the mean (SE). Statistical significances of the differences between the means of the four groups of samples were assessed using one-way analysis of variance (ANOVA), followed by the Tukey's test. Differences were considered to be significant when *p *< 0.05.

## Results

**Figure 1:**
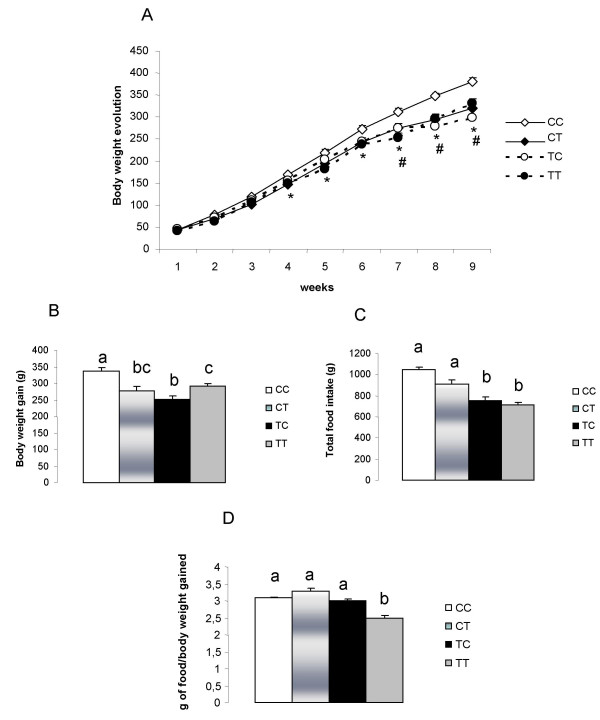
**(A) Body weight. (B) Total body weight gain. (C) Total food intake. (D) metabolic efficiency**. CC, mothers fed C diet and offspring fed C diet; CT, mothers fed C diet and offspring fed T diet; TT, mothers fed T diet and offspring fed T diet; TC, mothers fed T diet and offspring fed C diet. The number of studied animals per each group is 10. Data are means ± SE of 10 determinations per group. *p < 0.05, TT compared with CC. ^#^*p *< 0.05, TC compared with CC.

The body weights of the pups at 21 days old did not differ between the C and T groups. However, both the group TC at the seventh week and the group TT at the fourth week after weaning (Figure 1A) had lower body weights compared with CC. The total body weight gain in CC was higher than TT, TC and CT (Figure 1B). Also, TT had increase in total body weight gain as compared to TC group (Figure 1B). However, the food intake of both TT and TC was lower than CC and CT (Figure 1C).

The metabolic efficiency was higher in the TT group in relation to the other groups: for each gram of body weight gained, the TT ingested around 2.5 g of diet, whereas the other groups ingested around 3.2 g of diet (Figure 1D). The food intake of groups TC and TT was lower than CC (Table 3).

**Table 3:** Food intake (in grams per week)

Week	CC	CT	TC	TT
1^a^	67.3 ± 3.1^a^	58.3 ± 2.8^ab^	64.7 ± 3.0^a^	49.9 ± 1.4^b^
2^a^	108.3 ± 3.2^a^	91.7 ± 5.8^a^	72.9 ± 4.7^b^	94.3 ± 1.8^a^
3^a^	127.12 ± 5.4^a^	119.8 ± 5.2^a^	91.7 ± 5.9^b^	75.5 ± 4.1^b^
4^a^	158.4 ± 6.0^a^	120.3 ± 9.1^b^	100.3 ± 4.5^b^	101.6 ± 4.5^b^
5^a^	141.5 ± 5.71^a^	129.1 ± 6.9^ab^	113.0 ± 3.5^bc^	100.5 ± 3.8^c^
6^a^	153.9 ± 6.3^a^	132.8 ± 6.2^ab^	117.0 ± 5.1^b^	123.3 ± 5.4^b^
7^a^	145.1 ± 6.7^a^	141.0 ± 6.1^a^	96.57 ± 4.2^b^	137.3 ± 6.3^a^
8^a^	140.7 ± 4.3^ac^	117.1 ± 7.5^ab^	108.6 ± 4.7^b^	141.5 ± 6.1^c^

CC, mothers fed C diet and offspring fed C diet; CT, mothers fed C diet and offspring fed T diet; TT, mothers fed T diet and offspring fed T diet; TC, mothers fed T diet and offspring fed C diet. Data are means ± SE of 10 determinations per group. *p *< 0.05. Values in the same line with different superscript letters are significantly different from one another at *P *< 0.05.

**Figure 2:**
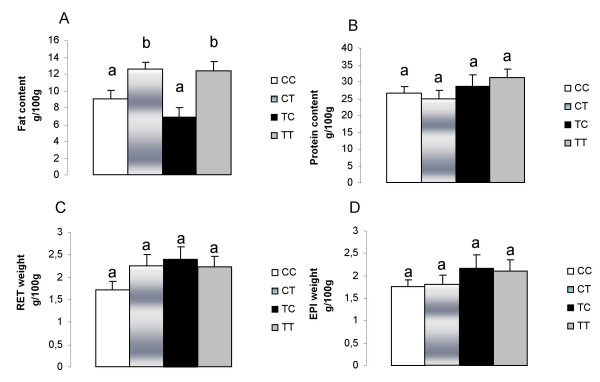
**(A) Carcass fat content. (B) Carcass protein content. (C) RET weight. (D) EPI weight**. CC, mothers fed C diet and offspring fed C diet; CT, mothers fed C diet and offspring fed T diet; TT, mothers fed T diet and offspring fed T diet; TC, mothers fed T diet and offspring fed C diet. Data are means ± SE of 10 determinations per group. *p *< 0.05.

The exposure to TFA after weaning (namely CT and TT groups) caused an approximate 40% increase in the body fat content (Figure 2A), however, the other corporal parameters measured (body protein content, RET and EPI relative weight) were no different among the groups (Figure 2).

Glycemia, lipid profile and leptin blood levels were similar among the studied groups. However, adiponectin blood levels were increased in TT (by 69%) and TC (by 34%) groups and insulinemia was higher in TT (by 84%) compared with the control group (Table 4).

**Table 4:** Serum glucose, triglycerides, total cholesterol, HDL-cholesterol, leptin, adiponectin and insulin

	CC	CT	TT	TC
Glucose (mg/dl)	118.7 ± 4.7^a^	98.8 ± 6.3^a^	108.4 ± 2.0^a^	101.8 ± 2.0^a^
Triglycerides (mg/dl)	125.0 ± 13.5^a^	126.0 ± 15.8^a^	166.5 ± 11.6^a^	147.9 ± 12.2^a^
Cholesterol (mg/dl)	106.6 ± 11.5^a^	110.5 ± 9.5^a^	107.9 ± 8.4^a^	125.9 ± 5.8^a^
HDL-cholesterol (mg/dl)	55.2 ± 6.3^a^	62.4 ± 5.7^a^	53.4 ± 3.9^a^	66.0 ± 6.0^a^
Cholesterol/HDL-cholesterol	1.9 ± 0.05^a^	1.8 ± 0.04^a^	2.0 ± 0.1^a^	2.0 ± 0.1^a^
Leptin (ng/ml)	10.10 ± 0.77^a^	8.80 ± 0.67^a^	9.23 ± 1.01^a^	7.18 ± 1.46^a^
Adiponectin (μg/ml)	51.33 ± 4.71^a^	51.23 ± 4.32^a^	86.53 ± 3.79^b^	68.60 ± 6.37^c^
Insulin (ng/ml)	1.23 ± 0.20^a^	1.95 ± 0.39^b^	2.26 ± 0.25^b^	1.80 ± 0.34^ab^

CC, mothers fed C diet and offspring fed C diet; CT, mothers fed C diet and offspring fed T diet; TT, mothers fed T diet and offspring fed T diet; TC, mothers fed T diet and offspring fed C diet. Data are means ± SE of 10 determinations per group. *p *< 0.05. Values in the same line with different superscript letters are significantly different from one another at *P *< 0.05.

**Figure 3:**
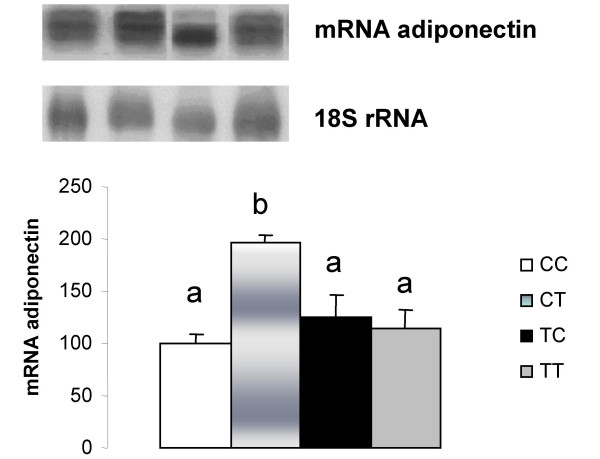
**Adiponectin mRNA quantification in retroperitoneal white adipose tissue**. CC, mothers fed C diet and offspring fed C diet; CT, mothers fed C diet and offspring fed T diet; TT, mothers fed T diet and offspring fed T diet; TC, mothers fed T diet and offspring fed C diet. Data are means ± SE of six determinations per group. Results are expressed in arbitrary units, stipulating 100 as the control value. *p *< 0.05.

**Figure 4:**
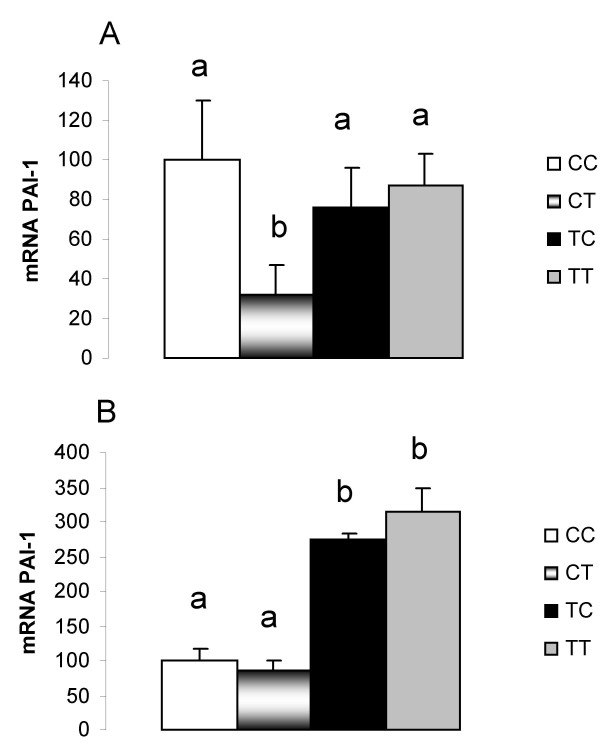
**PAI-1 mRNA quantification in (A) retroperitoneal and (B) epididymal white adipose tissues**. CC, mothers fed C diet and offspring fed C diet; CT, mothers fed C diet and offspring fed T diet; TT, mothers fed T diet and offspring fed T diet; TC, mothers fed T diet and offspring fed C diet. Data are means ± SE of six determinations per group. Results are expressed in arbitrary units, stipulating 100 as the control value. *p *< 0.05.

Adiponectin gene expression in RET of the CT group was higher than in the CC group (Figure 3). On the other hand, a decrease was observed in PAI-1 gene expression in the CT group (Fig 4A) compared with the other groups.

**Figure 5:**
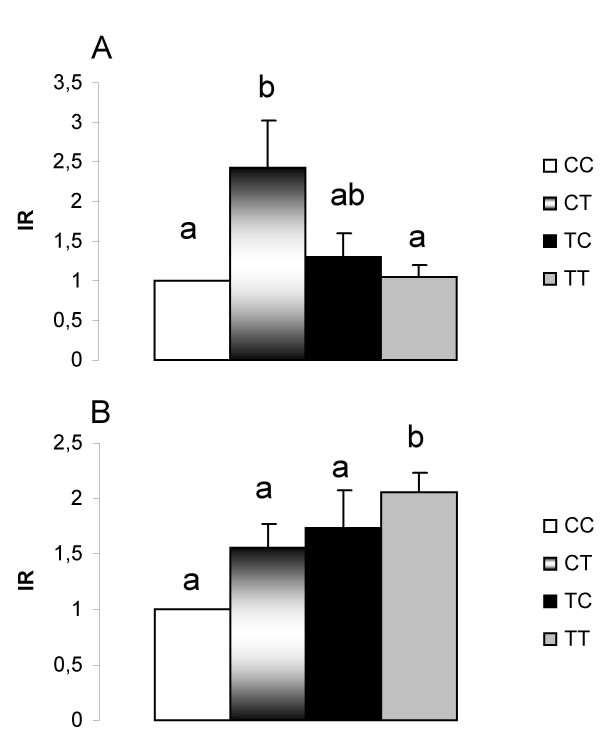
**Quantification of insulin receptor in (A) epididymal and (B) subcutaneous white adipose tissues**. CC, mothers fed C diet and offspring fed C diet; CT, mothers fed C diet and offspring fed T diet; TT, mothers fed T diet and offspring fed T diet; TC, mothers fed T diet and offspring fed C diet. Data are means ± SE of six determinations per group. Results are expressed in arbitrary units, stipulating 1 as the control value. *p *< 0.05.

PAI-1 gene expression in EPI was increased in the TT and TC groups (215% and 175% higher, respectively) compared with the CC and CT groups (Figure 4B). In this tissue, a higher protein level of insulin receptor (IR) was found in the CT compared with the CC and TT groups (Figure 5A).

Thus, treatment with the T diet from gestation to the end of the period studied (group TT) caused an increase in IR protein levels in SUB (Figure 5B).

## Discussion

In this study, the effects of maternal ingestion of hydrogenated vegetable fat rich in TFA, during gestation and lactation, followed by continued exposure of the offspring to this diet after weaning until the 90th day of life were investigated. We have also analyzed the effects of exposure to this diet just after weaning.

The food intake of the TC and TT groups was lower than the CC group, demonstrating that the ingestion of TFA by the mother during gestation and lactation can affect the feeding behavior of the offspring from 21 to 90 days of life.

Albuquerque et al [6] suggested that early exposure to TFA promoted adaptations in the hypothalamic mechanisms controlling food intake, and that these adaptations probably became programmed.

Wang et al [27] verified that the treatment with a high-fat diet rich in n-6 polyunsaturated fatty acids (PUFA) increased the gene expression of neuropeptide Y (NPY) in the arcuate nucleus of mice, as compared with mice treated with a high-fat diet rich in saturated fatty acids.

Since, the T diet contained TFA and saturated fatty acids and a low amount of n-3 and n-6 PUFA, in comparison with the control diet, it is possible to suggest that the TC and TT groups present low production of NPY and, as a consequence, hypophagia in relation to the control group. Likewise, Albuquerque et al [6] verified that the maternal ingestion of TFA reduced the concentrations of arachidonic acid, docosahexaenoic acid and total PUFA content in the total lipids of the brain obtained from 21-day-old offspring.

The ingestion of TFA reduced body weight gain. However, the TT group had the highest metabolic efficiency. These results suggest that the treatment, from fetal life until adulthood, with a diet rich in TFA and saturated fatty acids can reduce energy expenditure. Supporting this suggestion, Takeuchi et al [28] demonstrated that diet-induced thermogenesis is high in rats treated with a PUFA-rich diet compared with a diet rich in saturated fatty acids.

The carcass lipid content in the CT and TT groups was higher than in the CC group. Similar findings were described by Takeuchi et al [28] in animals treated with a diet rich in saturated fatty acids. Furthermore, Shillabeer and Lau [29] demonstrated that diets rich in saturated fatty acids promote the replication of adipocytes. It is possible that this mechanism contributed to the high carcass lipid content found in the CT and TT groups.

In a previous study, our group detected a dyslipidemia in the 21-day-old offspring of mothers treated with TFA [30], although the lipid profile was similar among groups in the 90-day-old rats used in the present study. Gutman et al [31] reported that the increased plasma triglyceride levels caused by the ingestion of high amounts of sucrose is multiphasic and depends on the period of treatment. This treatment was found to increase triglyceridemia progressively until the 22nd to 25th day of treatment, reduces to normal levels at the 45th to 50th day, and increases again at the 75th to 90th day of treatment. In this sense, it was demonstrated that rats fed with diets rich in TFA and saturated fatty acids for 12 weeks presented with increase serum insulin, triglyceride, and cholesterol levels [10]. These findings support our results and thus we cannot rule out the possibility that metabolic processes adapt to changes in the dietary components in a specific period of life.

Furthermore, a high adiponectin serum level was found in the TT and TC groups, which could be considered as a normal lipid profile, since it was described previously that adiponectin stimulates fatty acid oxidation and adiponectin serum level negatively correlates with triglyceride serum level [32].

The TT group had normoglycemia, increased insulin serum levels, and protein levels of IR in SUB. It has been shown that increased adiponectin levels are associated with a better glucose-level control in diabetics [33]. It is therefore reasonable to suggest that the increased serum adiponectin level associated with hyperinsulinemia and IR protein levels could contribute to the maintenance of glucose serum levels in the TT group.

The increase in PAI-1 gene expression in adipocytes was associated with insulin resistance [34] and hyperinsulinaemia [35]. This could partially explain the increases in PAI-1 mRNA (215%) in EPI of the TT group. However, we also found an increase in PAI-1 mRNA levels in EPI of the TC group compared with the CC group. Previously, we found high PAI-1 mRNA levels in 21-day-old offspring of rats fed a diet containing hydrogenated vegetable fat during gestation and lactation [30]. These results suggested that early exposure to hydrogenated vegetable fat caused an alteration in adipose tissue PAI-1 gene expression and that this alteration became programmed. Enhanced expression of the PAI-1 gene in visceral fat correlates with increased plasma levels [36] and a positive correlation between PAI-1 levels and cardiovascular disease is well described in the literature [11,37].

In summary, in this study we have found increased levels of insulin, adiponectin, body fat, and epididymal adipose tissue PAI-1 mRNA levels in 90-day-old offspring of rats fed a diet containing hydrogenated vegetable fat during gestation and lactation and exposed to the same diet after weaning (TT group). The *trans *offspring exposed to the control diet after weaning (TC group) also had an increase in adiponectin serum concentration and PAI-1 mRNA levels in EPI. These alterations were not observed in the CT group. It has been hypothesized that these conditions are produced by programming resulting from early exposure to dietary hydrogenated fat which could promote deleterious consequences, even after the withdrawal of the causal factor. TFA were probably important determinants of these alterations. However, it cannot be ruled out that differences in saturated and essential fatty acid contents between the control and the TFA diets played a role.

## Competing interests

The author(s) declare that they have no competing interests.

## Authors' contributions

LPP made substantial contributions to conception and design, experimental analysis and acquisition of data and also the analysis and interpretation of data. CMON participated in the design of the study and performed the statistical analysis and helped to draft the manuscript. AAB carried out the immunoassays and helped to draft the manuscript. CB participated in all molecular and biochemical analyses. KTA participated in the design of the study and in the insulin receptor analysis. EBR participated in the design of the study and performed the statistical analysis. LMO made substantial contributions to the conception and design, analysis and interpretation of the data and coordination to draft the manuscript. All authors read and approved the final manuscript.
